# Mechanical Properties of Three-Dimensional Printed Provisional Resin Materials for Crown and Fixed Dental Prosthesis: A Systematic Review

**DOI:** 10.3390/bioengineering10060663

**Published:** 2023-05-31

**Authors:** Saeed J. Alzahrani, Maher S. Hajjaj, Amr Ahmed Azhari, Walaa Magdy Ahmed, Hanin E. Yeslam, Ricardo Marins Carvalho

**Affiliations:** 1 Department of Restorative Dentistry, Faculty of Dentistry, King Abdulaziz University, Jeddah 21589, Saudi Arabia; 2 Department of Oral Biological and Medical Science, Faculty of Dentistry, The University of British Columbia, Vancouver, BC V63 1Z3, Canada

**Keywords:** dental materials, materials testing, computer-aided design, computer-aided manufacturing, printing

## Abstract

The emergence of digital dentistry has led to the introduction of various three-dimensional (3D) printing materials in the market, specifically for provisional fixed restoration. This study aimed to undertake a systematic review of the published literature on the Mechanical Properties of 3D- Printed Provisional Resin Materials for crown and fixed dental prosthesis (FDP). The electronic database on PubMed/Medline was searched for relevant studies. The search retrieved articles that were published from January 2011 to March 2023. The established focus question was: “Do provisional 3D-printed materials have better mechanical properties than conventional or milled provisional materials?”. The systematically extracted data included the researcher’s name(s), publication year, evaluation method, number of samples, types of materials, and study outcome. A total of 19 studies were included in this systematic review. These studies examined different aspects of the mechanical properties of 3D-printed provisional materials. Flexural Strength and Microhardness were the frequently used mechanical testing. Furthermore, 3D-printed provisional restorations showed higher hardness, smoother surfaces, less wear volume loss, and higher wear resistance compared to either milled or conventional, or both. 3D-printed provisional resin materials appear to be a promising option for fabricating provisional crowns and FDPs.

## 1. Introduction

The emergence of computer-aided design and computer-aided manufacture of CAD/CAM in dentistry facilitates the production of definitive crowns and FDP in a timely manner [1]. Unless the definitive prosthesis is delivered in the same visit, patients must be provided with a provisional restoration from the initial tooth preparation until the definitive prosthesis is placed [2]. Provisional restorations are an essential step in fixed prosthodontic treatment [3]. They are designed to enhance esthetics, function, and assess the effectiveness of a specific treatment plan [4]. Multiple clinical scenarios require a provisional phase, such as full mouth rehabilitation, immediate loading for implants, and crown lengthening cases [5,6].

Basic requirements for provisional restorations can be divided into biological, biomechanical, and aesthetic requirements [3]. They should be biocompatible, non-irritant, have a pleasant odor and taste, and provide a highly polished surface [4]. They must be strong, durable, hard, wear-resistant, and able to withstand the functional forces of mastication without fracture or displacement [4,7]. There are many methods, techniques, and materials to fabricate the provisional materials [8]. According to the chemical composition of provisional materials, they can be classified into two main groups [9]. The first group is acrylic resin based, which includes Polymethylmethacrylate (PMMA) and Polyethyl or butyl methacrylate (PEMA). The second group is composite resins based, such as urethane dimethacrylate (UDMA) and bisphenol A-glycidyl dimethacrylate (Bis-GMA).

With the emergence of digital dentistry, provisional materials can be classified according to the method of fabrication into two main categories: conventional or digital techniques [3,10]. The advantages of conventional techniques of fabrication of provisional materials include easy manipulation (hand mixing or an automix gun) mixing of the material and pouring it into a mold of the final shape of the desired prosthesis [11]. However, they are time-consuming, either chairside by clinicians or in the laboratory with dental technicians. In addition, acrylic resin-based materials might cause pulpal irritation from the exothermic reaction produced during the polymerization of resin material [4]. While composite resin-based materials are more brittle than acrylic resin [12].

Digital technology is used for creating temporary dental restorations. The process involves taking a detailed scan of the patient’s mouth or cast using an intraoral or desktop scanning method. Computer-aided design (CAD) software is then used to engineer the prosthesis with accuracy and precision. The final prosthesis is produced using either subtractive manufacturing ((SM) milling) or additive manufacturing ((AM) 3D printing) technology [13]. Subtractive manufacturing technology (milling) in the form of CAD/CAM [14,15,16]. Milling is a manufacturing process wherein the material is removed from the object from a prefabricated block or discs [17,18]. This technique of production has multiple disadvantages, such as the wasting of raw material, the limited use of milling tools in terms of numbers of units due to wear and abrasion, and the precision of the milled material being determined by the movement, type, and size of the tool used, which can lead to an inferior fit and marginal adaptation for complex designs [13,19].

However, 3D printing is a manufacturing process for building the intended object by incremental layering [20]. Recently, AM technology, or 3D printing, emerged in the literature due to its ability to overcome most of the disadvantages of milling [21]. In addition, the quality of 3D printing is continuously improving, which increases the usage of printing products and is expected to be even more so in the upcoming years, which includes their use in complex cases and with special needs patients [22,23,24,25]. The advantage of 3D printing over milling is to create restorations with fine detail of internal geometries or complex prostheses and reduce material waste [26,27].

There are multiple additive manufacturing methods using 3D printing in dentistry [28]. The most common methods for 3D printing are stereolithography (SLA), selective laser sintering (SLS), fused deposition modeling (FDM), digital light processing (DLP), polyjet, and bioprinting [29]. The printing mechanism of SLA printers relies on depositing resin from the vat [30,31]. SLS printers are used for printing different materials such as metals, plastics, and ceramics [32]. SLS and SLA printers both use lasers to create objects, but the distinction lies in the type of material they use. SLS employs powder-based materials, while SLA utilizes liquid-based materials [33]. DLP printers utilize a liquid-based printing material similar to SLA, but the curing process is accomplished through a light beam [34,35]. DLP printers are used to print models, surgical guides, dentures, occlusal guards, and wax patterns for casting [36]. FDM printers are limited to thermoplastic materials, which deposit heated drops of material fused to the previous hardened layer [37]. Polyjet printers utilize UV light to cure liquid photopolymers, allowing for the printing of complex objects with layers as thick as 25 micromillimeters [38]. The utilization of bioprinting has been recently integrated into the fields of tissue engineering and pharmaceutical applications [39,40].

Printing in dentistry has been limited to the fabrication of diagnostic casts, implant surgical guides, and occlusal guards [22,36]. Recently, many manufacturers have introduced different printing materials to the market for provisional fixed restoration. Many studies have investigated the mechanical properties of the available 3D-printed fixed provisional materials. In addition, limited systematic reviews have been conducted on the mechanical properties of 3D-printed fixed provisional resin materials [41,42]. Conducting a systematic review that summarizes the current status will save the time and effort of many restorative dental professionals interested in learning or using 3D-printed provisional materials as a helpful guide in daily clinical practice. Conducting this systematic review would provide a clear, unbiased comparison between all three fabrication methods (conventional, milled, and 3D-printed) for provisional fixed prostheses. 

Therefore, this study aimed to undertake a systematic review of the published literature on the mechanical properties of 3D-printed provisional materials for crowns and FDP. We conducted this review to test the null hypothesis that there was no significant difference in mechanical properties between 3D-printed provisional prostheses materials and the currently available milled and conventional provisional fixed materials. The underlying patient or problem, intervention, comparison, and outcome (PICO) question was: “Within the available studies in the literature, do provisional 3D-printed fixed materials have mechanical properties comparable to conventional or milled provisional fixed materials?”. The primary outcome was the mechanical properties of 3D-printed provisional fixed materials compared to conventional and milled resin materials. The secondary outcome was the mechanical properties variation between the different 3D-printed provisional materials and conditions.

## 2. Materials and Methods

Ethical approval was not applicable in the current study because it was exclusively based on the published literature and strictly adhered to research ethics by only using previously published studies ethically approved by original researchers [43,44]. 

### 2.1. Data Items

The well-known PICO strategy was used to select the focus question of this study. The PICO strategy used was as follows:

(1) Population: Dental provisional crown, bridge, or FDP.
(2) Intervention: 3D-printed provisional, interim, or temporary materials.
(3) Comparison: Milled or conventional fixed provisional or temporary or interim materials.
(4) Outcome: Mechanical properties.

The PICO focus question of the presented review was: “Within the available studies in the literature, do provisional 3D-printed fixed materials have mechanical properties comparable to conventional or milled provisional fixed materials?”. The null hypothesis was that 3D-printed provisional prostheses material mechanical properties are not different from the current available milled and conventional provisional fixed materials.

### 2.2. Information Sources and Search Strategy

The used research protocol, which was registered in the research registry (reviewregistry1599), implemented a systematic approach to searching the literature for relevant studies, screening selected studies for eligibility, and assessing their quality. The reporting of this systematic review was guided by the standards of the Preferred Reporting Items for Systematic Reviews and Meta-Analyses (PRISMA 2020) statement [45,46]. According to Page et al. in 2021 [29], PRISMA 2020 would not assess the quality of systematic reviews; however, familiarity and guidance by its checklist and instructions are crucial to conducting a systematic review that completely captures all recommended information. The reporting of PRISMA in the submitted systematic review follows suit of recently published systematic reviews that also reported guidance by the PRISMA 2020 guidelines without conducting a meta-analysis [47,48,49]. 

PubMed/Medline was electronically searched using Boolean operators to locate appropriate articles from January 2011 to March 2023 (Table 1). 

The titles and abstracts identified through the electronic search were exported into a Microsoft Excel sheet. The reference list of the initially included studies was manually searched for any additional relevant studies. The relevant articles were identified, and non-relevant articles were removed. 

### 2.3. Eligibility Criteria 

The included criteria were in vitro and in vivo studies that investigated mechanical properties with sufficient sample sizes and statistically analyzed results. They should be published in peer-reviewed journals available in English. Studies evaluating the mechanical properties of 3D-printed fixed provisional materials compared to conventional and/or milled provisional fixed materials. The exclusion criteria were review articles, case reports/series, study evaluation properties other than mechanical properties, not mentioning the material name, 3D-printed material used for removable prostheses such as the denture base, denture teeth, or occlusal/night guard prosthesis, non-dental uses of 3D-printed material, and studies without control samples (conventional or milled).

### 2.4. Study Selection and Data Collection Processes

Titles and abstracts were evaluated and selected for appropriateness by two independent investigators (AAA, WMA). Upon identification of a potential abstract, the full text of the article was retrieved. Articles were reviewed and subjected to inclusion and exclusion criteria. Any discrepancies after critical appraisal of the selected articles were resolved by discussion between the two investigators. 

### 2.5. Synthesis of the Results

A reading grid was used for data extraction, and the data were summarized in a table form. The systematically extracted data included the author/year of publication, evaluation method, types of materials (Conventional/Milled/printed), and study outcome. 

### 2.6. Data Extraction, Quality, and Bias Analysis

Cohen’s Kappa was calculated after performing the reviewers’ calibration. The cutoff value was set at 85%. By evaluating the risk of bias using criteria modified from earlier studies on basic research publications [50], quality evaluation was carried out. The set of requirements included: (1) Published materials in peer-reviewed journals. (2) Provides statistical analysis. (3) Randomization of test groups or controls. (4) Blinded evaluation. (5) Estimating the sample size before the experiment. (6) Examining the dose-response relationship. (7) A declaration of conformity to regulatory standards. (8) Objective congruence between the research and the relevant study. Depending on whether or not the parameters were reported, the quality level varied from low to high, reflecting our confidence that the estimation of the effect was correct. A positive choice with a score of 6 to 8 was deemed to have a low risk of bias, while one with a score of 3 to 5 was deemed to have a moderate risk of bias. A choice that was 1–2 positives was deemed to have a high bias risk.

## 3. Results

### 3.1. Study Selection

**Figure 1:**
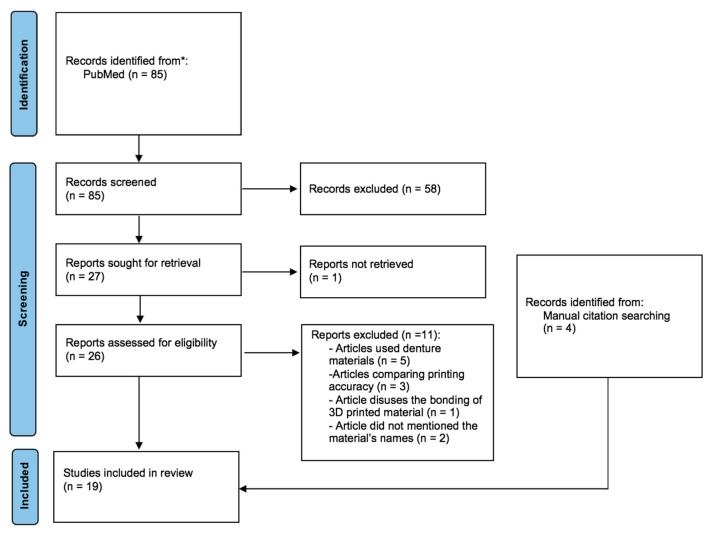
Flow chart of the study.

A total of nineteen studies were included in this systematic review. Fifteen studies were retained out of eighty-five studies, and four articles were manually added from the reference list of the retained articles. A flow chart of the selection process guided by the PRISMA 2020 statement was formulated. Figure 1 illustrates the process of identification of the relevant articles in a flowchart.

### 3.2. Quality of Selected Papers and Bias Reporting

Table 2 represents the outcome of the risk of bias assessment used in this systematic review. 

Most of the studies were published in the last 3 years. Out of the nineteen included studies, fourteen articles showed a low level of bias, and five articles showed a moderate level of bias. None of the included articles reported blindness of the investigator during the fabrication of the specimens or testing. Moreover, specimen randomization was only applied in nine studies. In addition, sample power calculation before testing was only mentioned in five articles. 

### 3.3. Study Characteristics

During data extraction, heterogeneity was observed in the methods adopted, the technique of fabrication of the samples, the materials used, and the parameters studied. These studies investigated different aspects of the mechanical properties of 3D-printed provisional resin materials, with a tendency to focus on the evaluation of fractural strength (FS), wear resistance (WR), surface roughness (SR), and hardness (VH and KH) properties. 

### 3.4. Results of Individual Studies

The results of individual studies were summarized in Table 3. 

### 3.5. Results of Synthesis, Data Analysis of the Mechanical Properties

It was found that 3D-printed provisional resin materials had a lower flexural strength (FS) compared with the milled provisional restorations [53,59,61,62,64,70]. However, 3D-printed provisional resin materials had a higher flexural strength when compared with the conventional provisional [52,54,56,57,61,65]. Wear resistance (WR) was the second common test used by investigators. Furthermore, 3D-printed materials showed generally higher wear resistance than both conventional and milled provisional materials [62,63,67,68,70]. Moreover, 3D-printed material generally had a higher Elastic Modulus (EM) Conventional, but it was not higher than the milled [53,54,55]. Hardness was evaluated by four studies using Knoop Hardness (KH) or Vickers Hardness (VH) with contradictory results [62,63,67,68,70]. Regarding surface roughness, the 3D-printed specimens displayed smoother surfaces than milled and conventional provisional materials [62,64,68,70]. Other mechanical properties, such as compressive strength, resilience, and toughness, were evaluated once in two different studies [57,59].

## 4. Discussion

Provisional restorations are an important step in fixed treatments, especially when inserting the final prosthesis during the same visit might be difficult. One of the recently adopted techniques for fabricating provisional crowns and FDP is 3D-printed provisional materials. This systematic review focused on evaluating the mechanical properties of the available 3D-printed resin materials and compared them to milled and conventional provisional resin materials. The null hypothesis that there is no significant difference in flexural strength, wear resistance, and surface roughness among the available 3D-printed fixed provisional materials was accepted. Furthermore, 3D-printed resin material showed generally good and comparable results to milled and conventional methods of fabrication of provisional materials.

In a recent systematic review in 2022, [41] mechanical properties excluding microhardness, toughness, and resilience, of 3D-printed provisionals were found to be superior to milled and conventional methods of fabrication of provisional crowns and FDPs. However, most of the pooled estimates included in that review were inconclusive, with very high heterogeneity, which restricted the applicability of the performed meta-analysis. Further investigation of factors affecting the mechanical properties was suggested [41]. In the current review, several factors were identified that influence the mechanical properties of 3D-printed materials: thickness of the printed layer, post-curing techniques, shrinkage between the layers, curing speed, intensity, angle, post-polymerization time and temperature, and printing direction [42]. Alharbi et al. [35] found that the compressive strength of 3D-printed materials improved by printing the layers perpendicular to the load direction. After printing two different provisional materials at 0, 45, and 90 degrees. Derban et al. [31] determined a significant impact of printing orientation on flexural strength.

A comparison of 3D printing resin mechanical properties with conventional and milled FDPs using three-unit FDP was performed by multiple studies [56,60,62]. There was an agreement of a higher flexural strength in milled provisional FDPs compared to conventionally fabricated provisional FDPs. The milled materials have higher fillers packed under higher temperatures and pressure, which result in lower porosity, voids, and residual monomers compared to 3D printing and conventional resins [62]. However, a printing orientation of 0° and 30° significantly improves the flexural strength of three-unit FDPs [60]. Despite the high variability between the two studies assessing the flexural strength of FDPs in terms of the post-curing process and cementation, milled FDP had a higher flexural strength compared to DLP FDP when using the same cleaning agent of isopropanol [62,65]. Investigating the effect of cementation is a crucial aspect, because cementation increases the FDP flexural resistance by evenly distributing the external forces throughout the specimen [3], and it is more clinically relevant than measuring the flexural strength without cementation.

All of the studies that evaluated flexural strength included in this systematic review had results higher than 50 MPa, which is the minimum flexural strength of the recommended fixed provisional prosthesis according to ANSI/ADA specification no. 27. The process of enhancing the flexural strength of printed polymers is still in progress. Aati et al. [29] modified the printable resin by adding zirconia oxide (ZrO_2_) nanoparticles at different concentrations. They found superior mechanical properties compared to the unmodified printable resin. 

Multiple studies that compared the wear resistance of 3D-printed materials showed less wear volume loss and displayed smoother surfaces compared to both milled and conventional provisional materials [62,63,67,68,70]. During printing, printers have the ability to deposit layers up to a tenth of a micromillimeter, which results in a product with a smoother surface and minimizes the polishing time in comparison to the milling [27]. There are many factors to be taken into consideration when evaluating wear tests. For instance, the machine used to perform the test, the media, the size and shape of the indenter, the load applied, and the number of cycles the samples undergo, are all factors that can create variabilities between the studies that affect our ability to quantitatively assess the data and draw a strong conclusion.

Hardness was evaluated by four studies with contrasting results [62,63,67,68,70]. Hardness was assumed to be higher in milled provisional resin materials due to higher cross-linked monomers and the presence of filler loading. Thus, increasing the hardness compared to conventional and 3D-printed materials. However, the SLA technique of printing had a comparable result to the milled technique [55]. This could be due to the printing technique mechanism itself, which allows printing successive layers under the controlled penetration of a UV laser beam [56].

There are still many concerns regarding 3D-printed materials that might influence their mechanical properties: cleaning the object of the remnants of uncured resin after the printing [62,71], and the ability to reline the shells and repair fractures [72,73]. The impact of post-curing techniques, the time taken, and the mechanical and physical accuracy of the 3D-printed materials [74,75,76]. Reymus et al. [76] found a degree of conversion influenced by the strategies of the curing used post printing, which will influence the mechanical properties of the material. Additionally, the maximum number of units for the fabrication of FDP and cleaning solutions must be thoroughly investigated before any recommendations can be made. 

This systematic review solely focused on the mechanical properties of 3D-printed materials and did not consider any other biological, chemical, or physical properties. Furthermore, the search methodology was restricted to published studies in the English language. Additionally, review articles in the Cochrane database, IEEE, and/or preprints were not included in the search, which could have limited the results. Another limitation is the fact that conducting and reporting a meta-analysis can be challenging due to significant differences between the included studies, making it difficult to combine and quantitatively analyze them. This heterogeneity can include indirect comparisons and studies that are too dissimilar to be combined and effectively analyzed [77] using different testing apparatuses, testing conditions, and/or specimen fabrication methods (crowns, FDP, implant crowns, bar-shaped and disc-shaped). Therefore, the authors suggest conducting additional studies that cover various aspects of the material properties and different clinical scenarios.

## 5. Conclusions

As more dental practitioners and technicians become interested in 3D-printed provisional materials, it is crucial to ensure that these materials have the best possible mechanical properties before they replace older methods such as milled and conventional provisional materials. Based on our systematic review, we have found that within its limitations, using 3D-printed provisional materials shows great promise for creating provisional crowns and FDP.

## Figures and Tables

**Table 1:** Search strategy terms and PICO in PubMed.

Search	Literature Search Strategy	Results
Population	Crown * OR exp Crowns/OR Fixed dental prosthesis OR bridge OR FDP OR Fixed partial prosthesis OR resin-bonded bridge OR bar OR specimen OR Dental Prosthesis Design* OR implant crown	778,817
Intervention	3D-printed provisional OR 3D-printed temporary OR 3D-printed interim OR 3D printed transient OR three-dimensional	283,947
Control	Conventional provisional OR Milling provisional OR PMMA OR polymethylemethacrylate OR Bis-acryl composite	19,315
Outcome	Mechanical properties OR flexural strength OR fracture toughness OR shear strength OR wear resistance OR chipping OR chip* OR surface roughness OR color OR color stability OR color alteration OR shade OR color change OR Bond* OR adaptation OR gap OR marginal discrepancy OR internal discrepancy	1,987,353
Total	(((Crown * OR exp Crowns/OR Fixed dental prosthesis OR bridge OR FDP OR Fixed partial prosthesis OR resin-bonded bridge OR bar OR specimen OR Dental Prosthesis Design* OR implant crown) AND (3D printed provisional OR 3D printed temporary OR 3D printed interim OR 3D printed transient OR three-dimensional)) AND (Conventional provisional OR Milling provisional OR PMMA OR polymeth-ylemethacrylate OR Bis-acryl composite)) AND (Mechanical properties OR flexural strength OR fracture toughness OR shear strength OR wear resistance OR chipping OR chip* OR surface roughness) 1 AND 2 AND 3 AND 4	85

**Table 2:** Risk of bias assessment tool according to Mikolajewicz et al. [50,51].

Author and Year	Peer- Reviewed	Complete stat	Randomization	Blindness	Sample Size Calculation	Dose-Response Relation	Statement of Compliance with Regulatory	Objective Aligned with Analysis	Risk of Bias
Sadek et al., 2023 [52]	+	+	-	-	+	+	+	+	Low
Bergamo et al., 2022 [53]	+	+	+	-	-	+	+	+	Low
Britto et al., 2022 [54]	+	+	+	-	-	+	+	+	Low
de Castro et al., 2022 [55]	+	+	+	-	-	+	+	+	Low
Ellakany et al., 2022 [56]	+	+	+	-	+	+	+	+	Low
Pantea et al., 2022 [57]	+	+	+	-	-	+	+	+	Low
Simoneti et al., 2022 [58]	+	+	+	-	-	+	+	+	Low
Tasin et al., 2022 [59]	+	+	-	-	+	+	+	+	Low
Turksayar et al., 2022 [60]	+	+	-	-	+	+	+	+	Low
Al-Qahtani et al., 2021 [61]	+	+	-	-	-	-	+	+	Moderate
Mayer et al., 2021 [62]	+	+	-	-	-	+	+	+	Moderate
Myagmar et al., 2021 [63]	+	+	+	-	-	+	+	+	Low
Martín-Ortega et al., 2021 [64]	+	+	+	-	-	+	+	+	Low
Park et al., 2020 [65]	+	+	-	-	-	+	-	+	Moderate
Reeponmaha et al., 2020 [66]	+	+	+	-	+	+	+	+	Low
Kessler et al., 2019 [67]	+	+	-	-	-	+	+	+	Low
Park et al., 2018 [68]	+	+	-	-	-	+	+	+	Moderate
Tahayeri et al., 2018 [69]	+	+	-	-	-	+	+	+	Low
Digholkar el al, 2016 [70]	+	+	-	-	-	+	+	+	Moderate

+: positive assessment of the item; -: negative assessment of the item.

**Table 3:** Summary of included studies with types of testing methods, types of specimens, materials used, and main outcomes.

Ref	Testing Method	Type of Specimens	Technique of Fabrication/Material Used (Brand Name Used^®^)	Outcome
Sadek et al., 2023 [52]	FS	Disk-shaped (10 × 2-mm)	Conventional: -PMMA (Tempron) -DMA (Protemp), -DMA (Tuff-Temp) Milled: -PMMA (VITA CAD-Temp) -PMMA (breCAM.multiCOM) Printed: - NextDent C&B (Nextdent B.V)	FS: printed ↑ than milled and conventional
Bergamo et al., 2022 [53]	FS and EM	Bar-shaped (25 × 2 × 2 mm)	Conventional: -PMMA (Alike, GC) -PMMA (Dencor) -DMA (Tempsmart), -DMA (Yprov, Yller) Milled: -PMMA (TelioCAD) Printed: -CosmosTemp-DLP (Yller)	FS and EM: ↔ to 3D printed and ↓↓ than milled and DMA after TC. EM: ↓↓ than all except DMA.
Britto et al., 2022 [54]	FS and EM	Bar-shaped (25 × 2 × 2 mm)	Conventional: -PMMA (Coroas e Pontes), -DMA (Yprov Bisacryl) Printed: -CosmosTemp-DLP (Yller)	FS and EM: showed ↑↑ than conventional DMA.
de Castro et al., 2022 [55]	FS, EM and KH	Bar-shaped (25 × 2 × 2 mm) Disk-shaped (15 × 2.5-mm)	Conventional: -PMMA (VitaTemp, Vita) Printed: -CosmosTemp-SLA (Yller) -CosmosTemp-DLP (Yller) -PriZma-Bioprov (Makertech) -Nanolab 3D (Wilcos)	After 24-h load: FS and EM: ↓↓ all printed than milled After 1-year load: FS: ↓↓ Nanolab and DLP than milled. ↓↓ DLP at printing orientation 45° than 0°,and 90° ↔ SLA(45°) to milled, ↑ SLA(90°) than milled EM: ↓↓ all printed than milled KN: ↑↑ Nanolab than milled.
Ellakany et al., 2022 [56]	FS and VH	3-unit FDPs	Conventional: -PMMA (Unifast TRAD) Milled: -PMMA (TelioCAD) Printed: - NextDent C&B (Nextdent B.V) -ASIGA (Denta Tooth)	VH and FS: printed SLA ↑↑ than conventional and ↔ to milled. However, printed DLP ↓↓ than milled. EM: printed SLA ↓↓ than milled Fracture site: connector except SLA in pontic
Pantea et al., 2022 [57]	CS, FS and EM	Cylindrically-shaped (25 × 25 mm) Bar-shaped (80 × 20 × 5 mm)	Conventional: -PMMA (Duracryl) -PMMA (Superpont) Printed: - NextDent C&B (Nextdent B.V) -HARZ Labs Dental Sand (HARZ Labs)	FS and EM: ↑↑ printed than conventional -Printed more homogenous
Simoneti et al., 2022 [58]	FR, SR and VH	Crown Rectangular-shaped (4 × 2 ×10 mm) Disks-shaped (10 × 2 mm)	Conventional: -PMMA (Dencor) -DMA (Yprov Bisacryl) printed: -Stratasys-SLS (Stratasys) -Gray Formlabs (Formlabs)	VH: Acrylic resin ↑↑ values than printed FR: SLA resin having the ↓↓ SR: ↓↓ SLA and ↑↑ SLS resin.
Tasin et al., 2022 [59]	FS, resilience, and FT	Rectangular-shaped (25 × 2 × 2 mm)	Conventional: -PMMA (Temdent Classic) -DMA (Protemp 4) Milled -PMMA (Duo Cad) Printed -Temporis (DWS system).	FS: Milled ↔3D-Printed and ↓↓ Conventional.
Turksayar et al., 2022 [60]	FS	3-unit FDPs	Milled: -PMMA (Duo Cad) Printed: -Temporary CB (Formlabs)	FS: printing with 0° and 30° ↔ milled PMMA.
Al-Qahtani et al., 2021 [61]	FS, VH and SR	Bar-shaped (25 × 2 × 2 mm) Disk-shaped (3 × 10 mm)	Conventional: -PMMA (Jet) Milled: -PMMA (Ceramill) Printed: - Freeprint Temp (DETAX GmbH)	FS: printed ↑↑ than Conventional but ↔ than milled. VH and SR: printed ↑↑ than conventional and milled.
Mayer et al., 2021 [62]	FS and WR	3-units FDP	Milled -PMMA (Telio CAD) Printed -Temp PRINT (GC Europe) - NextDent C&B (Nextdent B.V) -Freeprint Temp (DETAX GmbH)	WR: Printed ↑↑ than Milled. FS: Printed ↓↓ than Milled.
Myagmar et al., 2021 [63]	WR and SR	Bar-shaped (15 × 10 × 10 mm) Rectangular-shaped (15 × 10 × 10 mm)	Conventional: - PMMA (Jet) Milled: -PMMA (Yamahachi Disk) Printed: -NextDent C&B (Nextdent B.V)	WR: Printed ↑↑ than convensional and milled. SR: Printed ↓↓ than convensional and milled
Martín-Ortega et al., 2021 [64]	FS	Implant’s crown	Milled: -PMMA (Vivo Dent CAD Multi) Printed: -SHERAprint-cb (Shera)	FS: printed ↓↓ than Milled
Park et al., 2020 [65]	FS	3-units FDP	Conventional: -PMMA (Jet) Milled: -PMMA (ViPi) Printed: - NextDent C&B (Nextdent B.V)	FS: Printed ↑↑ than Convensional but ↔ to milled.
Reeponmah et al., 2020 [66]	FS	Crown	Conventional: -PMMA (Unifast Trad) -MDA (Protemp 4) Milled: -PMMA (Prylic Solid) Printed: -Freeprint Temp (DETAX GmbH)	FS: ↔ between the study groups except convensional PMMA
Kessler et al., 2019 [67]	WR	Bar-shaped (NA)	Conventional: -DMA(Tetric EvoCeram) Milled: -PMMA (Telio CAD) Printed: -3Delta temp (Deltamed) -NextDent C&B (Nextdent B.V) -Freeprint Temp (DETAX GmbH)	WR: ↑ filler content of printed Materials showed ↑ WR
Park et al., 2018 [68]	WR	Bar-shaped (15 × 10 × 10 mm)	Conventional: -PMMA (Jet) Milled: -PMMA (ViPi) Printed: -NextDent C&B (Nextdent B.V)	WR: ↔ between the groups
Tahayeri et al., 2018 [69]	EM and PS	Bar-shaped (25 × 2 × 2 mm)	Conventional: -DMA (Integrity) -PMMA (Jet) Printed: -NextDent C&B (Nextdent B.V)	EM: Printed ↔ to PMMA but ↓↓ than DMA. PS: Printed ↔ to DMA but ↑↑ than PMMA. There was no direct correlation between printing layer thickness and EM or PS.
Digholkar et al, 2016 [70]	FS, WR	Bar-shaped (25 × 2 × 2 mm)	Conventional: -PMMA (NA) Milled: -PMMA (Ceramill) 3D-printed: -E-dent 100 (Envision TEC)	FS: printed ↓↓ among the groups WR: Printed ↑↑ among the groups

Key: ↑↑ = Significant Increase, ↑ = Increase, ↔ = No Significant Change, ↓↓ = Significant Decrease, FDP = Fixed Denture Prosthesis, FS = Flexural Strength, EM = Elastic Modulus, PS = Peak Stress, FM = Flexural Modulus, SR = Surface Roughness, WR = Wear Resistant, N/A = Not Applicable, PMMA = Polymethyl Methacrylate, DMA = Dimethacrylate, KH = Knoop Hardness, SLA = Stereolithography, DLP = Digital Light Processing, VH = Vickers Hardness, FT = Fracture Toughness, CS = Compressive Strength, CL = Cyclic Loading, FR = Fracture Resistance, SLS = Selective Laser Sintering, FR = Fracture Resistance, LCR = Light Cure Resin.
